# Supplementary material for: Linking individual and population patterns of rocky-shore mussels
Source: PeerJ. 2021 Dec 24;9:e12550. doi: 10.7717/peerj.12550 (PMC8711277; doi:10.7717/peerj.12550)
Supplement: Supplemental Information 1 — Table complementing Table 2. [file peerj-09-12550-s001.docx]

|  | | **Df** |  | **Mean sq** | **F** | **Value Pr(>F)** |
| --- | --- | --- | --- | --- | --- | --- |
| **BIOMASS** | |  |  |  |  |  |
| Intertidal height | | 1 |  | 2.428 | 0.2230 | 0.63898 |
| Shore | | 1 |  | 1.541 | 0.1416 | 0.70843 |
| Date | | 11 |  | 6.909 | 0.6347 | 0.78967 |
| Intertidal height*Shore | | 1 |  | 50.898 | 4.6762 | 0.03582 * |
| Date*Shore | | 11 |  | 10.216 | 0.9386 | 0.51329 |
| n = 72 | |  |  |  |  |  |
|  | **Log10 (DENSITY)** | | | | | |
| Intertidal height | | 1 | 0.12 | 0.09658 | 7.9245 | 0.0083 ** |
| Shore | | 1 | 0.086 | 0.55249 | 45.3340 | 3.656 x10^-08^ *** |
| Date | | 11 |  | 0.01574 | 1.2914 | 0.2891 |
| Intertidal height*Shore | | 1 | -0.05 | 0.00003 | 0.0022 | 0.9636 |
| Date*Shore | | 11 |  | 0.01008 | 0.7946 | 0.6443 |
| Residuals | | 46 |  | 0.01268 |  |  |
| n = 72 | |  |  |  |  |  |
|  | **MEDIAN LENGTH** (LMM, random factor: Date) | | | | | |
| Intertidal height | | 57 | 2.10 |  | 24.7736 | <0.0001*** |
| Shore | | 57 | 14.08 |  | 17.1512 | 0.0001*** |
| Intertidal height*Shore | | 57 | -2.47 |  | 1.6455 | 0.2048 |
| Observations n = 72  Groups n = 12 | |  |  |  |  |  |
|  | **MAX. LENGTH (LM)** | | | | | |
| Intertidal height | | 1 |  | 103.429 | 7.6602 | 0.008107 ** |
| Shore | | 1 |  | 61.044 | 4.5211 | 0.038878 * |
| Date | | 11 |  | 9.856 | 0.7299 | 0.704363 |
| Intertidal height*Shore | | 1 |  | 3.873 | 0.2869 | 0.594820 |
| Date*Shore | | 11 |  | 11.235 | 0.8321 | 0.609509 |
| Residuals | | 46 |  | 621.1 | 13.502 |  |
| n = 72 | |  |  |  |  |  |
|  | **CONDITION INDEX (GLM, family “gamma”, link “log”)** | | | | | |
| Intertidal height | | 1 |  |  | 1.1942 | 0.27487 |
| Shore | | 1 |  |  | 24.3377 | 1.013 x10^-06^ *** |
| Date | | 11 |  |  | 15.6995 | <2.2 x10^-16^ *** |
| Intertidal height*Shore | | 1 |  |  | 1.8838 | 0.17034 |
| Date*Shore | | 11 |  |  | 2.1452 | 0.01575 * |
| n = 720 | |  |  |  |  |  |
|  | **CROWDING INDEX** | | | | | |
| Intertidal height | |  |  | 0.0055 | 0.1267 | 0.7235 |
| Shore | |  |  | 0.0999 | 2.2907 | 0.1370 |
| Date | |  |  | 0.0382 | 0.8767 | 0.5686 |
| Intertidal height*Shore | |  |  | 0.1639 | 3.7552 | 0.0588 |
| Date*Shore | |  |  | 0.0486 | 1.1147 | 0.3720 |
| n = 72 | |  |  |  |  |  |
| **RECRUIT DENSITY** | |  |  |  |  |  |
| Adult Density (ind. m^-2^) | | 1 |  | 436355 | 16.3133 | 0.0002017 *** |
| Shore | | 1 |  | 1584 | 0.0592 | 0.8088363 |
| Date | | 11 |  | 131309 | 4.9090 | 5.478 x10^-05^ *** |
| Adult Density*Shore | | 1 |  | 6911 | 0.2584 | 0.6136704 |
| Date*Shore | | 11 |  | 13857 | 0.5180 | 0.8811617 |
| n = 72 | |  |  |  |  |  |
|  | **CROWDING INDEX** (LMM, random factor: Date) | | | | | |
| Adult Density | | 57 |  |  | 41.014 | <.0001* |
| Median length | | 57 |  |  | 29.670 | <.0001* |
| Shore | | 57 |  |  | 2.2983 | 0.1455 |
| Observations n = 72  Groups n = 12 | |  |  |  |  |  |
|  | |  |  |  |  |  |
