# Supplementary material for: Linking individual and population patterns of rocky-shore mussels
Source: PeerJ. 2021 Dec 24;9:e12550. doi: 10.7717/peerj.12550 (PMC8711277; doi:10.7717/peerj.12550)
Supplement: Supplemental Information 2 — Means sharing a letter are not significantly different based on the Tukey-adjusted comparisons. [file peerj-09-12550-s002.docx]

| Site | Date | mean CI | Group |
| --- | --- | --- | --- |
| East | Jan | 4.16 | abcd |
| East | Feb | 3.71 | ab |
| East | Mar | 3.59 | a |
| East | Apr | 4.34 | abcde |
| East | May | 4.89 | cdefg |
| East | Jun | 5.28 | efg |
| East | Jul | 4.90 | defg |
| East | Aug | 4.59 | bcdef |
| East | Sep | 5.04 | defg |
| East | Oct | 4.29 | abcde |
| East | Nov | 4.69 | cdefg |
| East | Dec | 4.79 | cdefg |
| West | Jan | 4.46 | abcdef |
| West | Feb | 4.18 | abcd |
| West | Mar | 3.92 | abc |
| West | Apr | 4.20 | abcd |
| West | May | 4.76 | cdefg |
| West | Jun | 5.27 | efg |
| West | Jul | 5.47 | fg |
| West | Aug | 5.72 | g |
| West | Sep | 5.48 | fg |
| West | Oct | 4.96 | defg |
| West | Nov | 5.10 | defg |
| West | Dec | 4.37 | abcde |
