# Supplementary material for: Linking individual and population patterns of rocky-shore mussels
Source: PeerJ. 2021 Dec 24;9:e12550. doi: 10.7717/peerj.12550 (PMC8711277; doi:10.7717/peerj.12550)
Supplement: Supplemental Information 3 — Significant differences between shores are indicated in exponent: non-significant p > 0.05: NS; p < 0.05: *; p < 0.01:**; p < 0.001: ***. Relationships between the crowding index, adult density and population median length combine west and east shores results, as represented in Fig. 8F. [file peerj-09-12550-s003.docx]

| Relationships between individual traits / population parameters and the intertidal height | | | | |
| --- | --- | --- | --- | --- |
|  | West shore | | East shore | |
|  | Intercept | Slope | Intercept | Slope |
| Log_10_(Mortality rate, Z) ~ Intertidal height (m) | -0.69* | 0.11 | -1.03* | 0.21 |
| Log_10_(density) ~ Intertidal height (m) | 3.98* | 0.12 | 3.97* | 0.07 |
| Median length (mm) ~ Intertidal height (m) | -0.54* | 2.10 | 13.54* | -0.38 |
| Maximal length (mm) ~ Intertidal height (m) | 57.39 | -4.64 | 63.92 | -5.57 |
| Relationships between population parameters | | | | |
| Recruit density (ind. m^-2^) ~ adult density (ind.m^-2^) | 43 | 0.011 | 160 | 0.0034 |
|  |  |  |  |  |
|  | Intercept | | Slope | |
| Crowding index ~ adult density (ind.m^-2^) (^a^) |  | 0.57 | 0.000029 |  |
| Crowding index ~ median length (mm) (^b^) |  | 0.85 | 0.036 |  |

(^a^) Intercept when the adult density is 2500 ind. m^-2^.

(^b^) Intercept when the median length is 12.5 mm.
