# Supplementary figures and images for: Linking individual and population patterns of rocky-shore mussels
Source: PeerJ. 2021 Dec 24;9:e12550. doi: 10.7717/peerj.12550 (PMC8711277; doi:10.7717/peerj.12550)

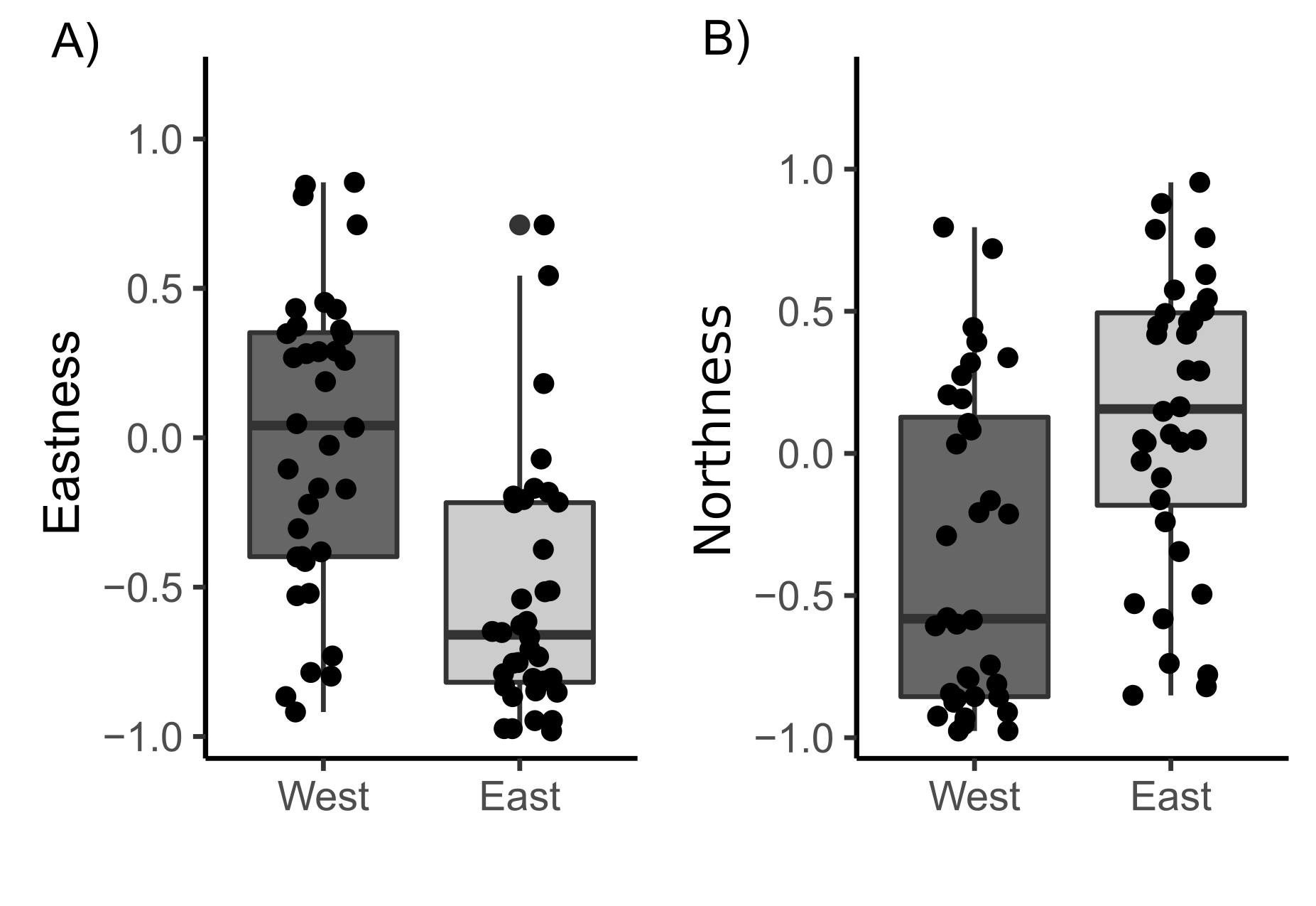

Supplement: Supplemental Information 4 — The lower and upper hinges of the boxplots correspond to the first and third quantiles, the middle line corresponds to the mean value, and whiskers (vertical lines) indicate the highest and smallest values (between 1.5 * the interquartile range (IQR)). Each point corresponds to one quadrant sample. [file peerj-09-12550-s004.png]
